# *Encephalitozoon cuniculi* and Extraintestinal Microsporidiosis in Bird Owners

**DOI:** 10.3201/eid2803.211556

**Published:** 2022-03

**Authors:** Marta Kicia, Żaneta Zajączkowska, Martin Kváč, Kamil Cebulski, Nikola Holubová, Piotr Wencel, Leszek Mayer, Maria Wesołowska, Bohumil Sak

**Affiliations:** Wroclaw Medical University, Wroclaw, Poland (M. Kicia, Z. Zajączkowska, K. Cebulski, M. Wesołowska);; Biology Centre of the Czech Academy of Sciences, České Budějovice, Czech Republic (M. Kváč, N. Holubová, B. Sak);; University of South Bohemia, České Budějovice (M. Kváč, N. Holubová);; Al Aseefa Falcon Clinic, Dubai, United Arab Emirates (P. Wencel);; Medical University of Gdansk, Gdansk, Poland (L. Mayer)

**Keywords:** *Encephalitozoon cuniculi*, microsporidiosis, parasites, exotic birds, immunocompetent, zoonoses, Poland, Czech Republic

## Abstract

We identified *Encephalitozoon cuniculi* genotype II parasites as a cause of extraintestinal microsporidiosis in 2 owners of birds also infected with *E. cuniculi*. Patients experienced long-lasting nonspecific symptoms; the disease course was more progressive in a patient with diabetes. Our findings suggest direct bird-to-human transmission of this pathogen.

Microsporidia of the genus *Encephalitozoon* (*E. cuniculi*, *E. hellem*, and *E. intestinalis*) are intracellular pathogens infecting a wide range of animal species. Because spores can be released to the environment via hosts’ feces, urine, and respiratory secretions, they can be ingested or inhaled, posing a risk for zoonotic infection in humans (*1*). The primary site of *Encephalitozoon* spp. infection is the small-intestine epithelium, but dissemination and systemic infections are also well known. Infection of a broad spectrum of cell types has been noted, especially for *E. cuniculi* (*1*); various pathological changes affecting the digestive, urinary, and respiratory tracts and the nervous system may occur. Because encephalitozoons are opportunistic pathogens, the extraintestinal and disseminated infections and severe symptoms they cause are of concern in immunocompromised hosts, such as transplant recipients or persons living with HIV (*2*,*3*). Microsporidiosis develops in patients whose immune response has been weakened by diabetes or malignant disease treated with chemotherapy (*2*,*4*).

We describe the case of 2 bird owners in Poland who acquired *E. cuniculi*–caused microsporidiosis from their infected pet birds. The Human Research Ethics Committee of Wroclaw Medical University (Wroclaw, Poland) approved this study in accordance with agreement no. KB-549/2012. Patients provided written informed consent before examination.

## The Study

The 2 patients, a woman and a man, both 41 years of age, had nonspecific symptoms of fatigue, exhaustion, joint and muscle pain, frequent colds, and headaches, progressive and more severe in the woman. We observed fever reaching 38°C and lasting several months in both patients. Moreover, the woman had intense night palpitations, symptoms similar to bronchitis (occasionally treated with antimicrobial drugs), blurred vision, dizziness, and impaired concentration. She had had bipolar disorder and diabetes for years. Magnetic resonance imaging scans of her head revealed single minor demyelinating or vascular changes in the white matter of the frontal lobes. No information about diabetes treatment was available. No abnormalities were shown in abdominal ultrasound or chest radiograph. She tested seronegative for *Borrelia burgdorferi* and *Chlamydia psittaci* infection. Except for a low leukocyte count (3.00 cells/µL), the woman’s basic laboratory tests of blood and urine and her electrocardiograms showed no abnormalities.

Symptoms emerged in the patients after 2 years of breeding exotic birds together. They had 30 birds of various species: budgerigars, canaries, diamond doves, tricolored parrotfinches, Gouldian finches, and diamond firetails. During the 2-year period, 17 birds died from infectious and metabolic diseases, trauma, and management-related issues. 

As a part of standard flock management practice, we performed tentative postmortem diagnostic cytology in 1 tricolored parrotfinch; we suspected disseminated microsporidial infection and conducted a detailed investigation of pooled feces collected from 8 aviaries at the patients’ home and tissues from a dead budgerigar. All samples were delivered on ice to the Laboratory of Veterinary and Medical Protistology, Biology Centre of the Czech Academy of Sciences (České Budějovice, Czech Republic). We collected patients’ urine and stool specimens every 2 days for 1 week, 3 samples from each, during diagnosis and follow-up and examined them at the Department of Biology and Medical Parasitology, Wroclaw Medical University.

We homogenized all samples by mechanical disruption before genomic DNA extraction as described previously (*5*). We performed nested PCR protocols amplifying a partial sequence of 16S rRNA gene (130 bp), the entire internal transcribed spacer region, and a partial sequence of 5.8S rRNA gene (137–139 bp) of *Encephalitozoon* spp. (*6*). In addition, we checked for the presence of *Enterocytozoon bieneusi* (*7*) and then performed a phylogenetic analysis of PCR products (*5*). We used standard light microscopy methods for spore detection (*8*). We deposited the sequences obtained in this study in GenBank (accession nos. OK356650–60). 

We detected microsporidial DNA in the fecal specimens of all birds and in tissue samples from both tested birds. Genotyping revealed the presence of *E. hellem* genotype 1A in all tested specimens from the lung, liver, duodenum, and jejunum with ileum from the budgerigar, whereas *E. cuniculi* genotype II was found in pooled feces and in all sections from the liver, gizzard with proventriculus, and duodenum with jejunum and ileum from the tricolored parrotfinch. 

*E. cuniculi* genotype II was present in 2 of the woman’s urine samples and in 1 of the man’s urine samples (Figure). Spores were confirmed in all samples. *E. bieneusi* was not found. We administered 400 mg albendazole daily for 10 days to both patients. In follow-up examination 3 months after treatment, the patients’ urine repeatedly tested negative for *E. cuniculi* in all independent samplings, and the patients gradually improved. Patients’ stools remained negative during the entire diagnostic process. No subsequent follow-up was conducted.

**Figure:**
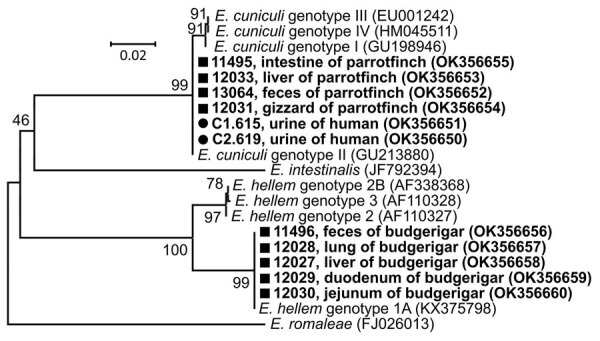
Phylogenetic relationships of *Encephalitozoon cuniculi* genotype II and *E. hellem* genotype 1A obtained from 2 exotic bird breeders and 2 of their birds compared with other *Encephalitozoon* species and genotypes. Bold type indicates sequences obtained in this study, identified by isolate number (e.g., C1.615); black circles indicate isolates from humans; squares indicate isolates from birds. We analyzed a partial sequence of 16S rRNA gene, the entire internal transcribed spacer region, and a partial sequence of 5.8S rRNA gene inferred by neighbor-joining analyses and computed using the Tamura 3-parameter method. We modeled the rate variation among sites with a gamma distribution. Percentages of replicate trees in which the associated taxa clustered together in the bootstrap test (1,000 replicates) are shown next to the branches. The final dataset contained a total of 220 positions. GenBank accession numbers are in parentheses. Scale bar indicates nucleotide substitutions per site.

## Conclusions

Birds are a common source in the propagation of encephalitozoons in the environment (prevalence <15%), either acting as mechanical vectors or developing active infection (*9*–*11*). In both wild-living and captive birds, *E. hellem* is the most prevalent *Encephalitozoon* species, whereas *E. cuniculi* has been detected less frequently (*9*,*10*). Whether animal hosts indeed propagate microsporidia or serve as transmission vectors is debatable (*9*). However, the presence of pathogens in both feces and tissues of birds in our study confirms microsporidial proliferation in these animals rather than the passage of spores through the digestive tract; this finding suggests that encephalitozoons may have been circulating in this breeding group for some time. Although most earlier reports demonstrated asymptomatic infections and low infection intensity among birds, intermittent spore shedding in naturally infected birds contaminates the environment (*11*). Owners could be in constant contact with spores, which highly increases the risk for infection by ingestion or inhalation of spores. As a slow-growing pathogen, *E. cuniculi* can lead to chronic infection and microsporidiosis.

In immunocompetent hosts, an immune-controlled balance in the host–parasite relationship is established, and extraintestinal infections remain asymptomatic (*5*). Symptomatic cases of microsporidial infection usually manifest as self-limiting diarrhea (*2*). Symptomatic extraintestinal *Encephalitozoon* infections in immunocompetent humans are uncommon and usually present as keratoconjunctivitis (*2*). Of note, disseminated microsporidiosis caused by *E. cuniculi* genotype I with involvement of brain and urinary and intestinal tracts has been described in men with type 2 diabetes (*4*). However, diabetes is considered a risk factor for opportunistic infections. Experimental infection of diabetic mice with *E. cuniculi* resulted in more symptoms and a higher pathogen burden than in nondiabetic animals (*12*); indeed, the woman with diabetes in our study experienced more severe symptoms, which led to serious impairments in everyday functioning.

Even though we were able to test only urine and feces and confirmed *E. cuniculi* in the urinary tract, the symptoms we observed in our patients were complex, indicating disseminated infection. We cannot be confident in the extent to which *E. cuniculi* infection contributed to these symptoms. However, symptom relief coincided with pathogen clearance after albendazole treatment, which convinced us that microsporidia may have been at least partially involved in symptom development. The severity of symptoms despite the lack of lifelong immunosuppression is puzzling and may arise from high doses of spores acquired as a result of patients’ everyday contact with birds.

In summary, our study documents the risk for bird-to-human transmission of *E. cuniculi* parasites. Exotic-bird breeders should be aware of the risk for infection with this opportunistic pathogen.
